# Strategies to Avoid Artifacts in Mass Spectrometry‐Based Epitranscriptome Analyses

**DOI:** 10.1002/anie.202106215

**Published:** 2021-09-29

**Authors:** Steffen Kaiser, Shane R. Byrne, Gregor Ammann, Paria Asadi Atoi, Kayla Borland, Roland Brecheisen, Michael S. DeMott, Tim Gehrke, Felix Hagelskamp, Matthias Heiss, Yasemin Yoluç, Lili Liu, Qinghua Zhang, Peter C. Dedon, Bo Cao, Stefanie Kellner

**Affiliations:** ^1^ Ludwig-Maximilians Universität München Butenandtstr. 5–13 81137 München Deutschland; ^2^ Department of Biological Engineering and Center for Environmental Health Sciences Massachusetts Institute of Technology Cambridge MA 02139 USA; ^3^ Ella Biotech GmbH 82152 Munich Germany; ^4^ College of Life Sciences Qufu Normal University Qufu Shandong 273165 China; ^5^ Antimicrobial Resistance Interdisciplinary Research Group Singapore-Massachusetts Institute of Technology Alliance for Research and Technology 138602 Singapore Singapore; ^6^ Institute of Pharmaceutical Chemistry Goethe-University Frankfurt Max-von-Laue-Str. 9 60438 Frankfurt Germany

**Keywords:** digestion artifact, mass spectrometry, nucleoside analysis, RNA modification, RNA PT

## Abstract

In this report, we perform structure validation of recently reported RNA phosphorothioate (PT) modifications, a new set of epitranscriptome marks found in bacteria and eukaryotes including humans. By comparing synthetic PT‐containing diribonucleotides with native species in RNA hydrolysates by high‐resolution mass spectrometry (MS), metabolic stable isotope labeling, and PT‐specific iodine‐desulfurization, we disprove the existence of PTs in RNA from *E. coli*, *S. cerevisiae*, human cell lines, and mouse brain. Furthermore, we discuss how an MS artifact led to the initial misidentification of 2′‐*O*‐methylated diribonucleotides as RNA phosphorothioates. To aid structure validation of new nucleic acid modifications, we present a detailed guideline for MS analysis of RNA hydrolysates, emphasizing how the chosen RNA hydrolysis protocol can be a decisive factor in discovering and quantifying RNA modifications in biological samples.

## Graphical abstract

For discovery of novel RNA modifications, a stringent workflow for structure validation is necessary to avoid misinterpretation of artifacts. The comparison of the synthetic standard to the native compound's retention time, full mass spectrum, high‐resolution mass spectrum and metabolic isotope labeling allow for confident identification of RNA modifications.

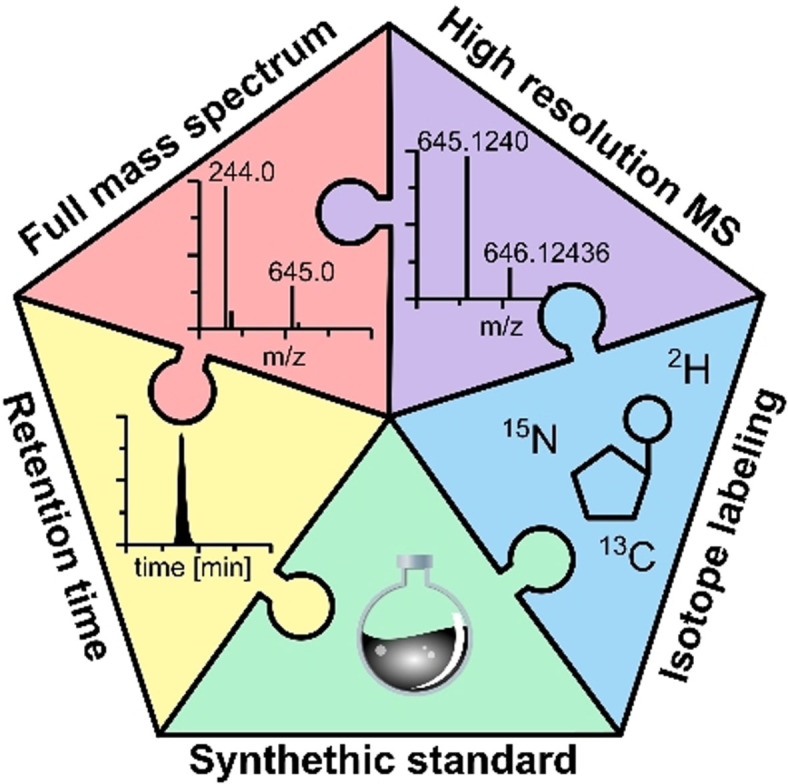

## Introduction

All forms of RNA are initially transcribed with four canonical building blocks, with the transcripts then being enzymatically decorated with any of more than 170 chemical modifications that define the epitranscriptome.^[1]^ The most recently proposed addition to the epitranscriptome family involves the first known modification of the phosphate backbone with substitution of a non‐bridging phosphate oxygen with sulfur as a phosphorothioate (PT) in both prokaryotes and eukaryotes.^[2]^

While PT modifications are new for RNA, they have previously been observed in bacterial DNA.^[3]^ The sulfur in the PT renders the nucleic acid vulnerable to oxidation, resulting in strand breaks.^[4]^ This instability has led to the initial observation of a sulfur‐containing DNA modification that caused strand breaks during electrophoresis,^[5]^ before the modification was characterized as a PT by mass spectrometry (MS).^[3]^ Furthermore, this property has now been exploited to determine the location of PTs in bacterial genomes at single‐nucleotide resolution through iodine‐induced cleavage and sequencing‐based mapping of the breaks.^[6]^ PTs are introduced into DNA by a specialized enzyme complex, DndABCDE,^[7]^ where DndA acts as a cysteine desulferase. In *E. coli*, DndA can be replaced by the desulferase IscS,^[8]^ which is involved in various bacterial RNA thiolation processes.^[9]^ Half of all PT‐containing bacteria have an additional set of restriction enzymes, DndFGHI, as part of a classical restriction‐modification system.^[10]^ In other bacteria, PTs are involved in an epigenetic interplay with the 6‐methyladenosine introduced by the DNA methyltransferase Dam.^[11]^ The genomic insertion of PT is beneficial to microorganisms and thus a wide distribution of PT in the human microbiome is not surprising.^[12]^

The discovery of most RNA modifications, including PT in RNA, has been facilitated by sensitive MS analysis. Although this approach is straight‐forward and new modifications are reported on a regular basis,^[13]^ the sensitivity of modern mass spectrometers and the need for multiple types of mass spectrometry for rigorous structural definition are potential pitfalls. Jora et al. showed that low abundance artifacts introduced by enzymatic RNA hydrolysis can be misinterpreted as novel RNA modifications.^[14]^ In the original RNA hydrolysis protocol by Crain and colleagues,^[15]^ the hydrolysis is performed in two steps, first at pH 5 using nuclease P1 (NP1) and phosphodiesterase 1 (PDE1) followed by dephosphorylation by alkaline phosphatase at pH 8. A one‐pot alternative using Benzonase instead of NP1 at pH 8 has been reported and is now widely used.^[16]^ At pH 8, the labile RNA modification cyclic N(6)‐threonylcarbamoyladenosine (ct^6^A) undergoes epimerization and various artifacts arise.^[17]^ Furthermore, not all enzymes used for RNA hydrolysis are capable of cleaving modified nucleotides. For example, nucleases S1 and P1 are not able to cleave m^7^G from the mRNA 5′‐m^7^GpppN cap, which has been exploited for cap analysis in transcripts.^[18]^ However, other nucleases, such as PDE1, can cleave the cap structure as well as RNA phosphodiester bonds to release m^7^G for analysis, which He and co‐workers exploited to differentiate m^7^G in caps from the body of mRNA.^[19]^

Given the large and growing variety of RNA modifications,^[1]^ there is growing pressure on researchers to correctly distinguish isobaric and structurally similar modifications as well as to rigorously identify new structures. Here we provide a guide for the discovery and structural validation of new nucleic acid modification candidates. We applied this approach to the recently described RNA phosphorothioate modification^[2]^ and found that the correct identity of the modification in the nuclease‐resistant diribonucleotide species is 2′‐*O*‐methylated ribose. Our systematic comparison of RNA hydrolysis protocols highlights the central role of the hydrolysis step and structural validation by high‐resolution MS and other methods in RNA modification discovery experiments as well as in absolute quantification of modified nucleosides.

## Results and Discussion

**Figure 1:**
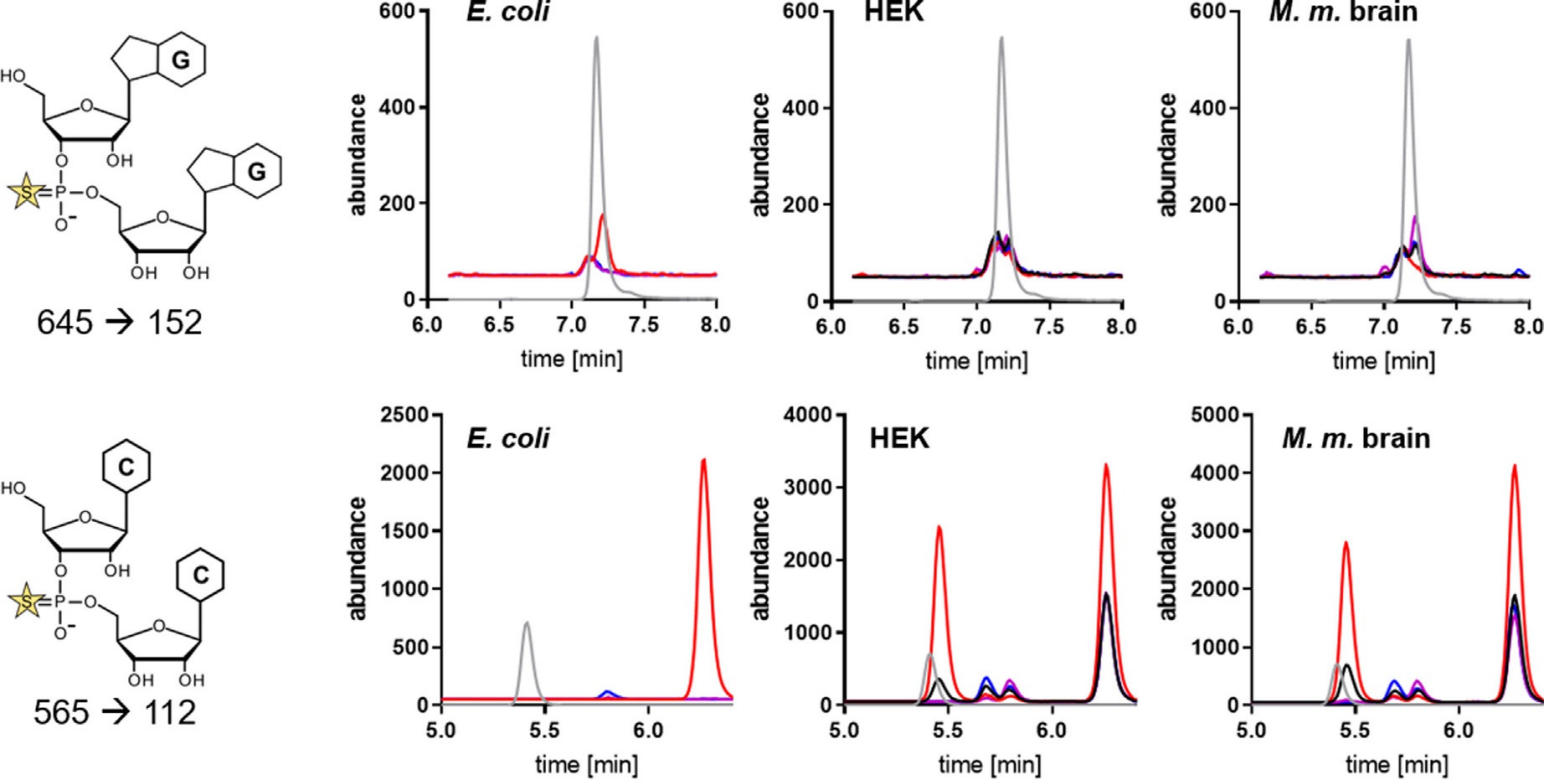
Signals for RNA phosphorothioates using precursor ion to product ion detection in targeted MS/MS analysis of RNAs from *E. coli* K12, HEK cells and mouse brain tissue. Upper row: GpsG analysis; lower row: CpsC analysis. The structure on the left shows the MS‐MS transition used to detect the PT‐containing diribonucleotide. LC‐MS tracings: grey, synthetic standards for Rp isomers of GpsG and CpsC; black, total RNA; red, tRNA; purple, small rRNA subunit (16S or 18S); blue, large rRNA subunit (23S or 28S).

Mass spectrometric analysis of DNA phosphorothioation depends on the hydrolytic stability of the PT towards several nucleases, including nuclease P1 (NP1). Nuclease treatment releases PT‐linked dinucleotides from the DNA and is exploited to quantify and characterize the dinucleotide context by LC‐MS.[^3^, ^4^] For synthetic PT‐containing RNA, we observe the same stability towards NP1 (Figure S1) and thus LC‐MS analysis of PT is possible by NP1 hydrolysis followed by detection of the PT‐linked diribonucleotide.^[2]^ Under the assumption that PTs might occur within any combination of canonical ribonucleosides, 16 possible PT diribonucleotide structures must be considered during method development. In addition, thiolation of the phosphate backbone introduces a stereocenter and thus Rp and Sp isomers of each dinucleotide must be established. With the goal of developing a fast and reliable method for absolute quantification of native PTs in RNA, all 32 possible Rp and Sp PT dinucleotides were prepared as reported^[2]^ and their HPLC retention times and MS characteristics assessed by LC‐MS/MS (Figure S2). We then analyzed total RNA from *E. coli* K12 and B7A strains, human embryonic kidney cells (HEK 293), and mouse brain tissue for the presence of PT‐containing diribonucleotides. Total RNA was first fractionated by size‐exclusion chromatography following established protocols^[20]^ to yield tRNA, 16S/18S small ribosomal RNA, and 23S/28S large ribosomal RNA. Each fraction was then hydrolyzed with NP1 and analyzed using the developed LC‐MS method. We observed signals similar to the synthetic PT precursor and product ions of GpsG and CpsC in all digests with variable abundance depending on the identity of the respective RNA fraction (Figure 1). While the signal for native GpsG and the synthetic Rp‐GpsG overlapped, we noticed several signals for CpsC in the various species, with only one of these overlapping with the synthetic Rp‐CpsC standard.

**Figure 2:**
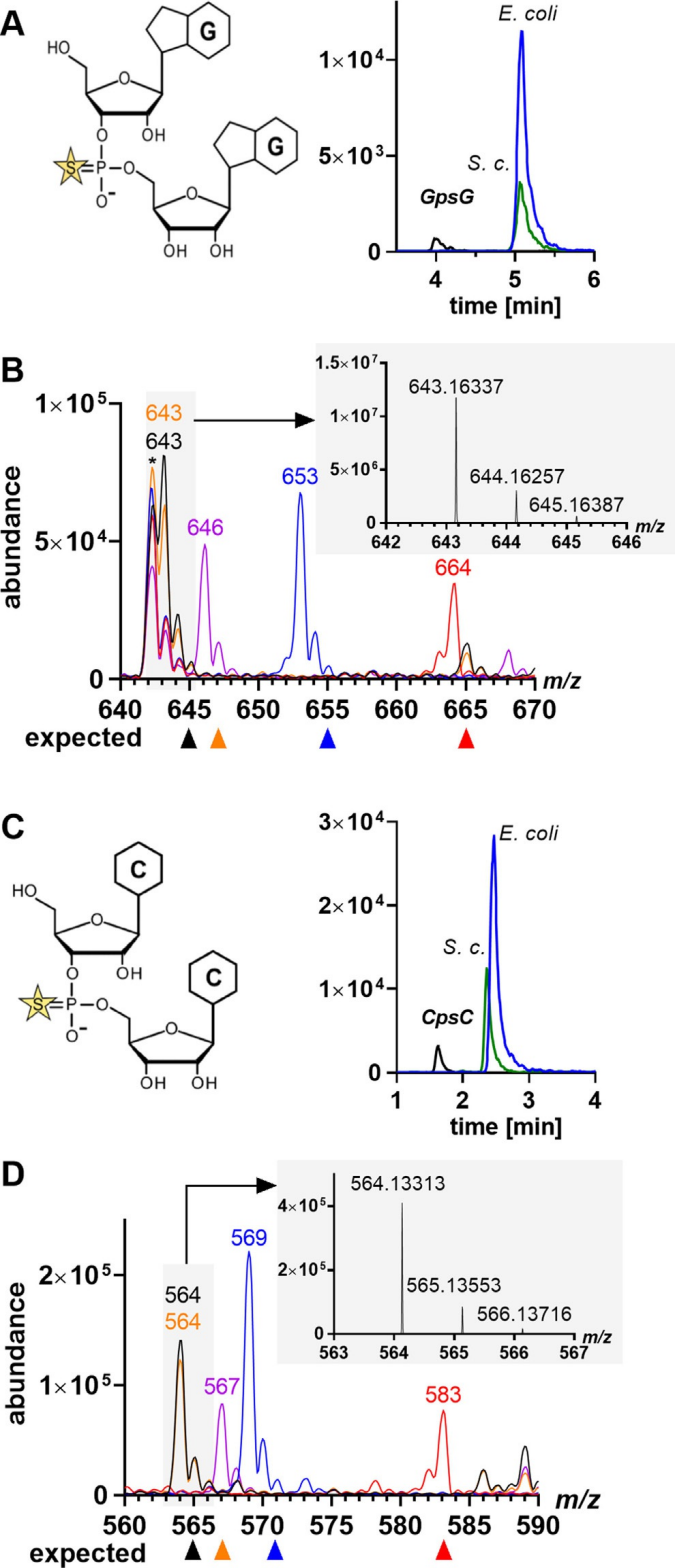
Comparison of synthetic GpsG and CpsC and native diribonucleotide signals. UHPLC‐MS/MS of synthetic (black) GpsG (A) and CpsC (C) and native RNA hydrolysates of *E. coli* B7A (blue) and *S. cerevisiae* (green). Mass spectra of stable isotope labeled RNA from *E. coli K12* of the PT diribonucleotide candidates GpsG (B) and CpsC (D). Color code to isotope labels: black—unlabeled; orange—^34^S; blue—^15^N; red—^13^C; and purple—L‐methionine‐[^2^H_3_]‐methyl. *Co‐eluting contaminant. Grey inset: High‐resolution mass spectra (HRMS).

The observation with CpsC merited a more detailed analysis of the native PT dinucleotide signals by orthogonal UHPLC‐MS/MS analysis using high‐resolution mass spectrometry (HRMS) and metabolic isotope labeling. UHPLC‐HRMS analysis with adapted solvent gradient of the RNA isolated from *E. coli* B7A and *S*. c*erevisiae* revealed a discrepancy in the retention times of the dinucleotides relative to the synthetic standards for CpsC and GpsG PT dinucleotides (Figure 2). The putative CpsC dinucleotide eluted 30 s more slowly than the synthetic Rp PT standard, while the putative GpsG dinucleotide eluted 60 s more slowly than the GpsG PT standard. These results suggested that the dinucleotides isolated from *E. coli* and *S. cerevisiae* were not PT‐containing dinucleotides.

To ascertain the identities of the observed dinucleotides present in RNA from these organisms, total RNA was extracted from both *E. coli* B7A and *S. cerevisiae* and digested to a mixture of ribonucleosides and diribonucleotides suspected to contain PT. The putative PT‐containing diribonucleotides were isolated by preparative HPLC for HRMS analysis. High‐resolution mass spectra were obtained by orbitrap mass spectrometry for both the synthetic PT diribonucleotide standards and the diribonucleotides isolated from biological samples, which revealed a 2 Da discrepancy (Figure 2). Unexpectedly, the exact mass found for the native GpsG signal is 1.96017 Da lighter compared to the exact mass observed for synthetic GpsG (Figures 2 B and S3A). In addition, synthetic GpsG showed the natural ^34^S signal (4 % at M+2), while the native GpsG did not. Metabolic stable isotope labeling of all carbon, nitrogen or sulfur atoms was performed in *E. coli* K12 using minimal medium M9 containing a single source for ^34^S, ^13^C and ^15^N respectively, and the RNA was purified and analyzed as described earlier.[^4^, ^13b^, ^21^] Human cells were stable isotope labeled by feeding ^15^N_5_‐adenine and/or ^15^N_2_^13^C_5_‐labeled uridine as recently reported.^[22]^ MS analysis of ^34^S‐labeled *E. coli* RNA showed an absence of sulfur in the native analyte, which is a strong indication against a PT diribonucleotide structure. However, after growing the cells with L‐methionine‐[^2^H_3_]‐methyl, a signal at *m*/*z* 646 indicated the presence of a methyl group in the diribonucleotide (Figure 2 B).^[13b]^ Complete ^15^N and ^13^C labeling in *E. coli* ^[13b]^ also does not provide evidence for the putative GpsG structure. Similar results were obtained in stable isotope labeled HEK cells (Figure S3A), where we observed a methylation mark after L‐methionine‐[^2^H_3_]‐methyl feeding (*m*/*z* 646). The mass increase from *m*/*z* 643 to 651 (+8 Da) indicates the presence of two ^15^N_4_‐labeled guanine bases, which confirms its nature as a canonical phosphate‐linked GG dinucleotide in HEK cells. The presence of two guanine bases is additionally supported by analysis of the ^15^N‐RNA extracted from *E. coli*, which is 10 Da heavier than the starting material with the exocyclic amino group labeled with ^15^N here in addition. From this data, we conclude that there is no evidence for GpsG in *E. coli*, *S. cerevisiae*, human cells or mouse brain tissue. In Figure 2 C, we focused on the multiple signals for CpsC obtained through targeted LC‐MS analysis of native RNA. HRMS of synthetic CpsC and native putative CpsC showed a mass discrepancy of 0.97811 Da (Figures 2 D and S3B). Again, stable isotope labeling provided evidence of a methyl group instead of a sulfur in the analyte. Furthermore, the mass difference between unlabeled and ^15^N‐labeled signals indicates the presence of only five nitrogen atoms, whereas CpsC has six. We analyzed the corresponding peak from isotope labeled HEK RNA and confirmed the presence of a methyl group and two pyrimidine ribonucleosides (due to the mass increase of +14, Figure S3B). In human and mouse RNA, five signals were found in targeted CpsC MS analysis using the chromatographic system from Figure 1 (Figure S3). The first signal co‐elutes with synthetic Rp‐CpsC, but HRMS analysis of this signal revealed a *m*/*z* of 564 and thus the same ≈1 u mass discrepancy as seen in *E. coli* and peak 4 of HEK cells in Figure S3. Similarly, the signal vanishes in the presence of L‐methionine‐[^2^H_3_]‐methyl and a 3 Da heavier signal at *m*/*z* 567 appears, which suggests the presence of a methyl group. The MS spectra from all other peaks from targeted CpsC analysis did not show the expected *m*/*z* of 565 for unlabeled RNA, while a signal for methylation can be found in all of them (Figure S3C).

**Figure 3:**
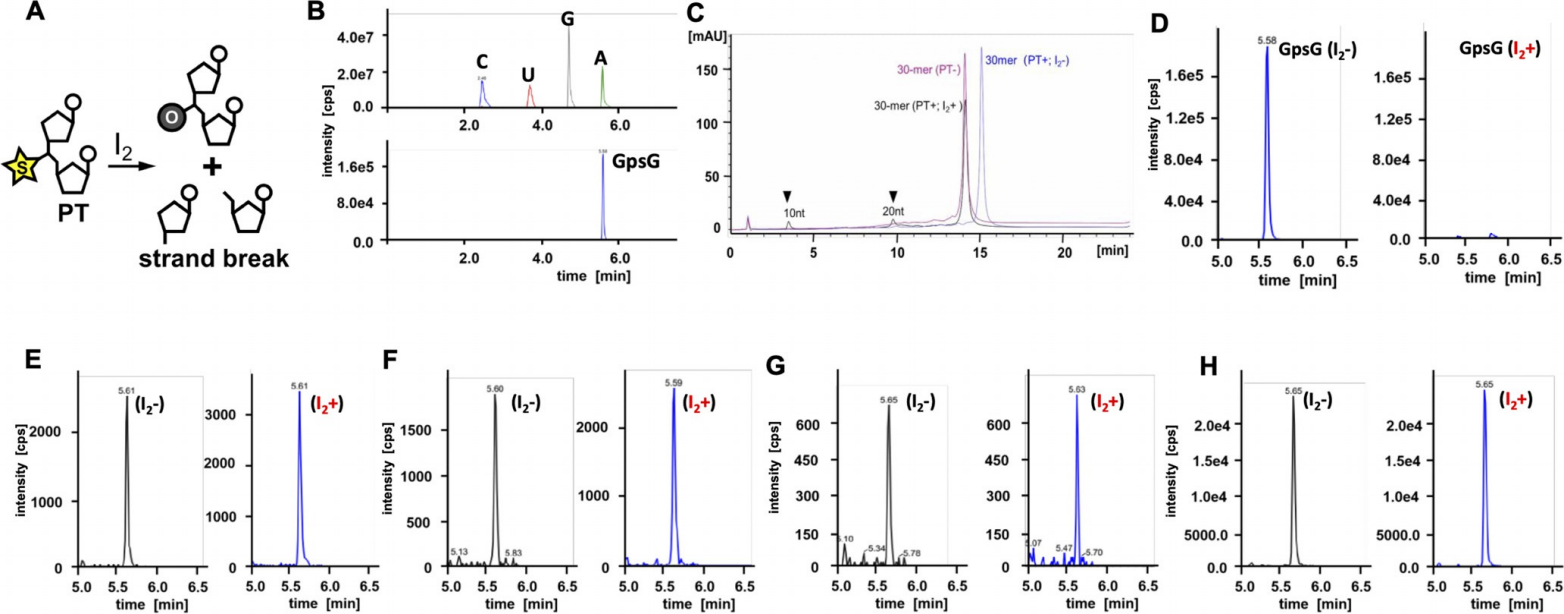
Detection of RNA phosphorylation by iodine cleavage. (A) Scheme showing iodine‐induced oxidation of a putative RNA PT modification. (B) UPLC‐MS/MS identification of the GpsG diribonucleotide in a 30‐mer synthetic RNA oligoribonucleotide. (C) HPLC analysis of the iodine‐treated 30‐mer RNA oligo reveals ≈20 % cleavage into 10 nt and 20 nt fragments and ≈80 % desulfurization to phosphate at the GpsG site. Blue: 30‐mer GpsG oligo; red: 30‐mer oligo lacking PT; black: 30‐mer PT‐containing oligo treated with iodine. (D–H) UPLC‐MS/MS analysis of the iodine‐treated 30‐mer RNA oligo reveals near complete loss of of GpsG diribonucleotide (D), while the co‐eluting putative “GpsG” is stable to iodine in the total RNA from *E. coli* B7A (E), *E. coli* B7A (*Δdnd BCDE*) DNA PT‐deficient mutant (F), *Saccharomyces cerevisiae* BY4741 (G) and human A549 cells (H).

The results obtained with multiple mass spectrometric approaches convincingly demonstrate that there are no PT‐containing diribonucleotides in RNA from four model organisms, with the most likely identity of the modified species being 2′‐*O*‐methylated dinucleotides. For the sake of rigor, we tested the presence of PTs in RNA by exploiting the sensitivity of PTs towards iodine oxidation (Figure 3 A). This has been used for PT‐specific cleavage and subsequent mapping of PT sites in microbial DNA by next‐generation sequencing.^[6a]^ To establish iodine‐induced cleavage of RNA PTs, we synthesized a 30‐mer RNA oligoribonucleotide with a site‐specific GpsG PT and established the presence of the GpsG by NP1 digestion and UPLC‐MS/MS analysis (Figure 3 B). The oligo was then treated with iodine and the reaction mixture analyzed by HPLC. As shown in Figure 3 C, iodine treatment resulted in the formation of two shorter fragments of 10 nt and 20 nt, which is consistent with cleavage at GpsG site by iodine. As we showed with DNA,^[3]^ iodine‐induced strand breaks only accounted for ≈20 % of the oligo degradation, with ≈80 % of the oligo converted to a faster eluting 30‐mer oligo that co‐eluted with synthetic 30‐mer lacking PT (Figure 3 C). This is consistent with iodine‐induced desulfurization of PT to phosphate.^[3]^ To establish loss of GpsG in the iodine‐oxidized RNA, we analyzed the digestion mixture by UPLC‐MS/MS, which confirmed the loss of GpsG PT‐containing diribonucleotide (Figure 3 D). This approach was then applied to total RNA from *E. coli* B7A, which possesses Dnd genes for PT insertion in DNA, an *E. coli* B7A mutant lacking the Dnd genes (*Δdnd BCDE*), *S. cerevisiae* BY4741, and human A549 cells. Following iodine oxidation, the NP1‐hydrolyzed total RNA was analyzed by UPLC‐MS/MS. As shown in Figures 3 E–H, the presumed MS signal of putative GpsG diribonucleotide was stable to iodine treatment (Figure 3 E–H). Furthermore, we did not observe iodine‐induced RNA cleavage when total RNA was analyzed on a Bioanalyzer, again suggesting the absence of PTs in RNA (Figure S4). In summary, our orthogonal approaches show no evidence for PTs in RNA in *E. coli*, *S. cerevisiae*, mice or humans.

**Scheme 1:**
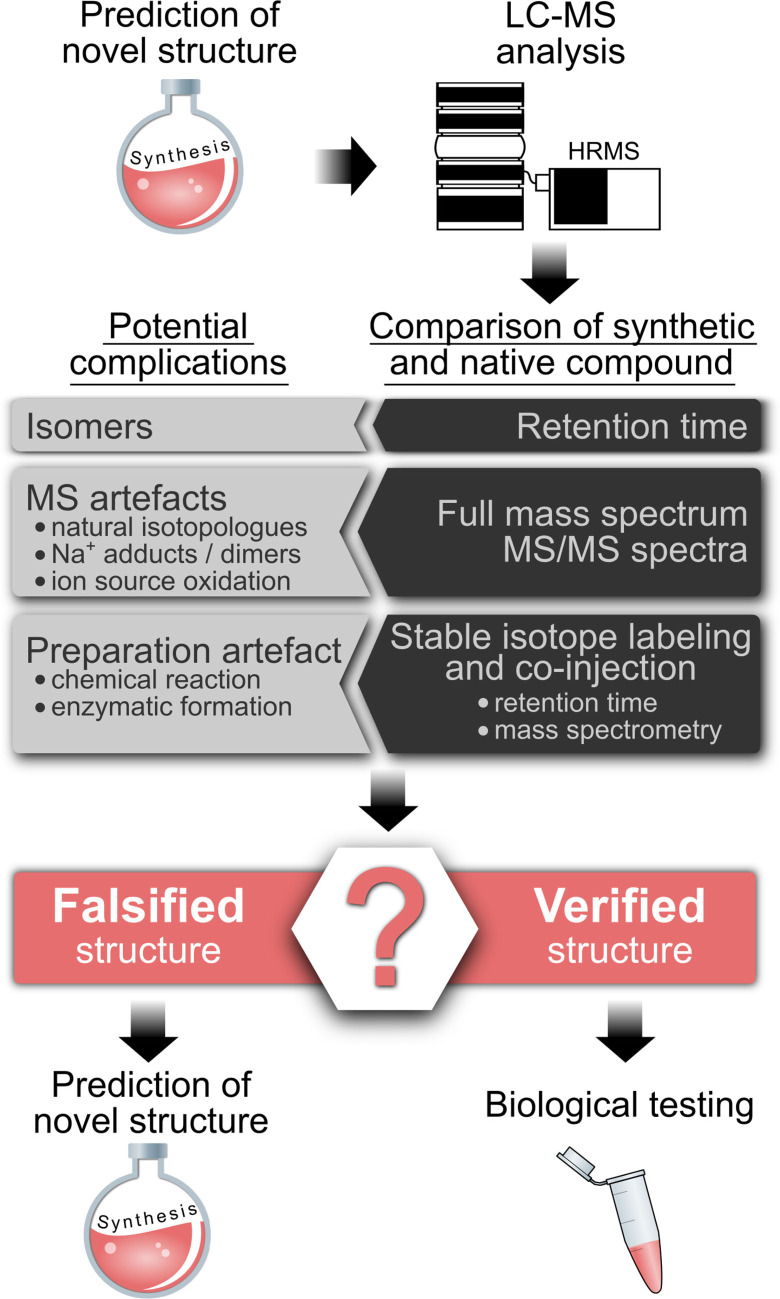
Approach to validating structures of DNA and RNA modifications.

The results of these studies cast doubt on the identity of the RNA‐derived molecules as PT‐linked dinucleotides, which initiates a process of predicting and proving the true structure. Here we refer to the workflow depicted in Scheme 1, which starts with a prediction of the structure. This can lead immediately to a metabolic isotope labeling study or, if a biosynthetic pathway can be predicted, a knockout or knockdown study to assess the modification level.[^13^, ^23^] In any event, the structure must be synthesized and compared to the native compound for behavior in LC‐MS and, if enough biological analyte exists, NMR studies. LC retention time represents a first dimension of identification and ideally more than one stationary/mobile phase pair is used to confirm co‐elution of synthetic and native compound. As a second dimension, a full mass spectrum of fragmentation on a high‐resolution instrument is required to establish exact molecular weight, MS/MS fragmentation patterns, and isotope envelopes. The chemical structure is confirmed if the native and synthetic versions behave identically.

However, one must also consider the possibility that the observed molecule is an artifact caused by adventitious enzymatic or chemical reactions during cell lysis, RNA purification, RNA processing, or even ionization in the mass spectrometer. Such artifacts are best excluded by analysis of stable isotope labeled nucleic acids. For example, aminations, which occur during some RNA hydrolysis protocols, are identified in ^15^N‐labeled RNA by the absence of one ^15^N.^[14]^ Furthermore, stable isotope labeled nucleic acids are ideal for co‐injection with the synthetic standard. Only compounds that pass this final step of structure validation should be taken into biological testing, including experiments on the compound's biosynthesis, location, distribution or quantity.

This strategy was applied here with a structural prediction that starts by considering that the MS analysis detected a signal at *m*/*z* 643 that was 2 Da lower in mass than the predicted GpsG, which should have had a signal at *m*/*z* 645. Considering that the predicted structure has 21 C atoms, the natural abundance of ^13^C (1.1 %) would produce an M+1 signal (*m*/*z* 644) that is 23 % of parent molecular ion (M) intensity and an M+2 signal (*m*/*z* 645) that is 2 % of M. The high sensitivity of triple‐quadrupole instruments can lead to a mistaken identification of M+1 or M+2 signals as M. The most immediately practical candidate dinucleotide structures that could account for this 2 Da difference are 2′‐*O*‐methylated dinucleotides, with 2′‐*O*‐methylated ribonucleosides occurring abundantly in most forms of RNA.

We tested this prediction in a series of studies that followed the checklist in Scheme 1.

**Figure 4:**
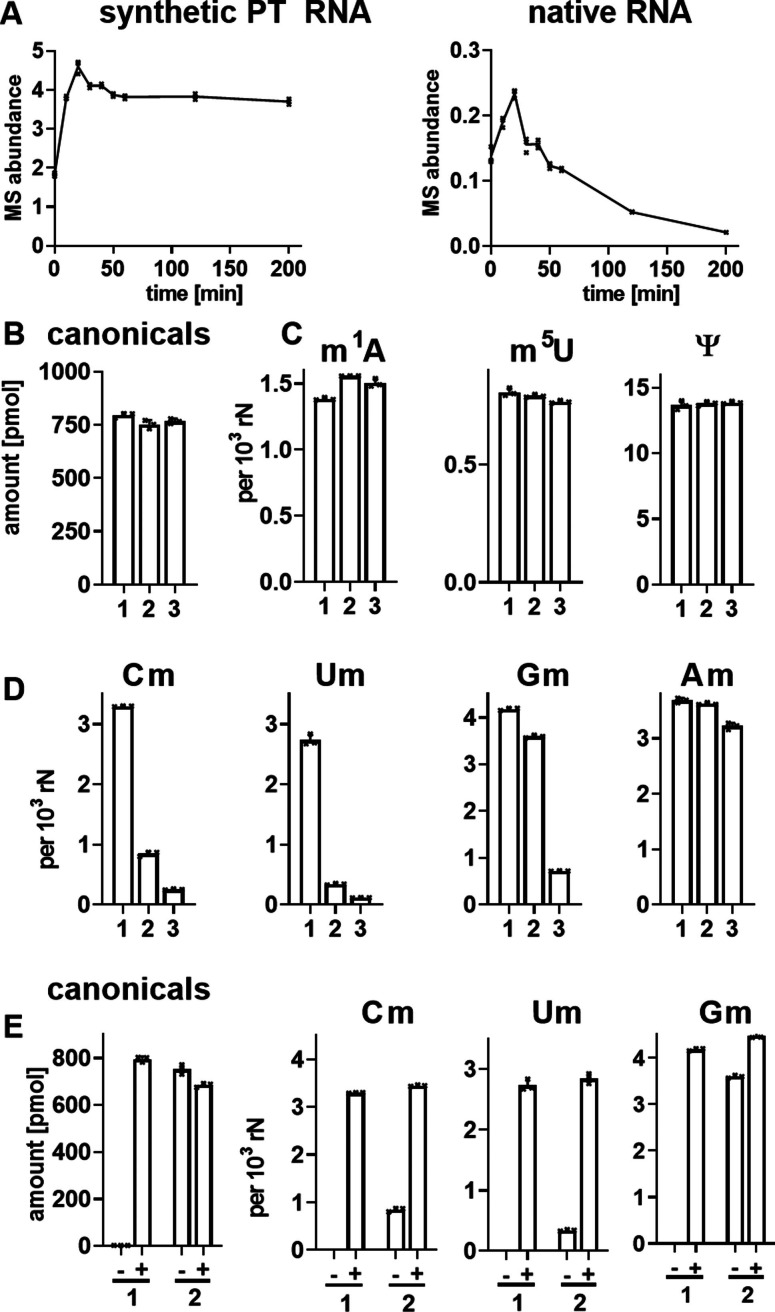
Impact of RNA hydrolysis conditions on detection of RNA modifications. (A) Abundance of peak with GpsG mass transition from synthetic GpsG containing RNA and native RNA from HEK cells digested with Nuclease P1 (NP1) and calf intestine phosphatase (CIP) at 37 °C for different incubation times. (B–D) Abundance of canonical nucleosides (rN) and various modified nucleosides from HEK total RNA digested with: 1, Benzonase/PDE1/CIP;^[16]^ 2, NP1/CIP;^[15]^ or 3 a commercial RNA hydrolysis kit (NEB, Nucleoside Digestion Mix). (E) Abundance of ribose methylated nucleosides from HEK total RNA digested in the absence (−) and presence (+) of phosphodiesterase 1 (PDE1) using either: 1, Benzonase+CIP^[16]^ or 2, NP1+CIP.^[15]^ All data represent mean ± SD for 3 experimental replicates.

We started by testing the 2′‐*O*‐methyl dinucleotide hypothesis as a sample preparation artifact: could 2′‐*O*‐methyl dinucleotides arise from incomplete hydrolysis of RNA? Indeed, more extensive hydrolysis of native RNA with NP1 for longer than 30 minutes decreases the dinucleotide signal. In contrast, dinucleotide signals from synthetic PT RNA are stable even after 3 hours of NP1 hydrolysis (Figure 4 A). This led us to compare common enzymatic RNA hydrolysis protocols for the completeness of the reaction. Here we used native RNA from HEK cells digested with either (1) Benzonase+phosphodiesterase I (PDE1)+calf intestine alkaline phosphatase (CIP) (protocol 1),^[16]^ (2) NP1+CIP^[15]^ (protocol 2; as in Figures 1 and 3), or (3) a commercial RNA hydrolysis kit (NEB, Nucleoside Digestion Mix), followed by quantification of the released nucleosides by isotope‐dilution LC‐MS/MS.

As shown in Figure 4 B, all three approaches release a similar amount of canonical ribonucleosides. Similarly, some modifications, such as 1‐methyladenosine (m^1^A), 5‐methyluridine (m^5^U) and pseudouridine (Ψ) are released in similar abundances (Figure 4 C). However, other modifications, especially the 2′‐*O*‐methylated ribonucleosides Cm, Um and Gm were detected at lower concentrations using protocol 2 and the kit (Figure 4 D), the latter also failing to release other modified nucleosides such as 5‐methylcytidine (m^5^C) (Figure S5). To understand why protocol 1 was superior to protocol 2, we repeated the experiment using Benzonase+CIP and NP1+CIP in the presence and absence of PDE1. As shown in the first graph of Figure 4 E, Benzonase alone does not fully hydrolyze RNA to the monoribonucleotide level for dephosphorylation by CIP. In contrast, NP1 produces a more extensive RNA hydrolysis in the absence of PDE1, but complete release of Cm, Um and Gm is only possible with the addition of PDE1.

**Figure 5:**
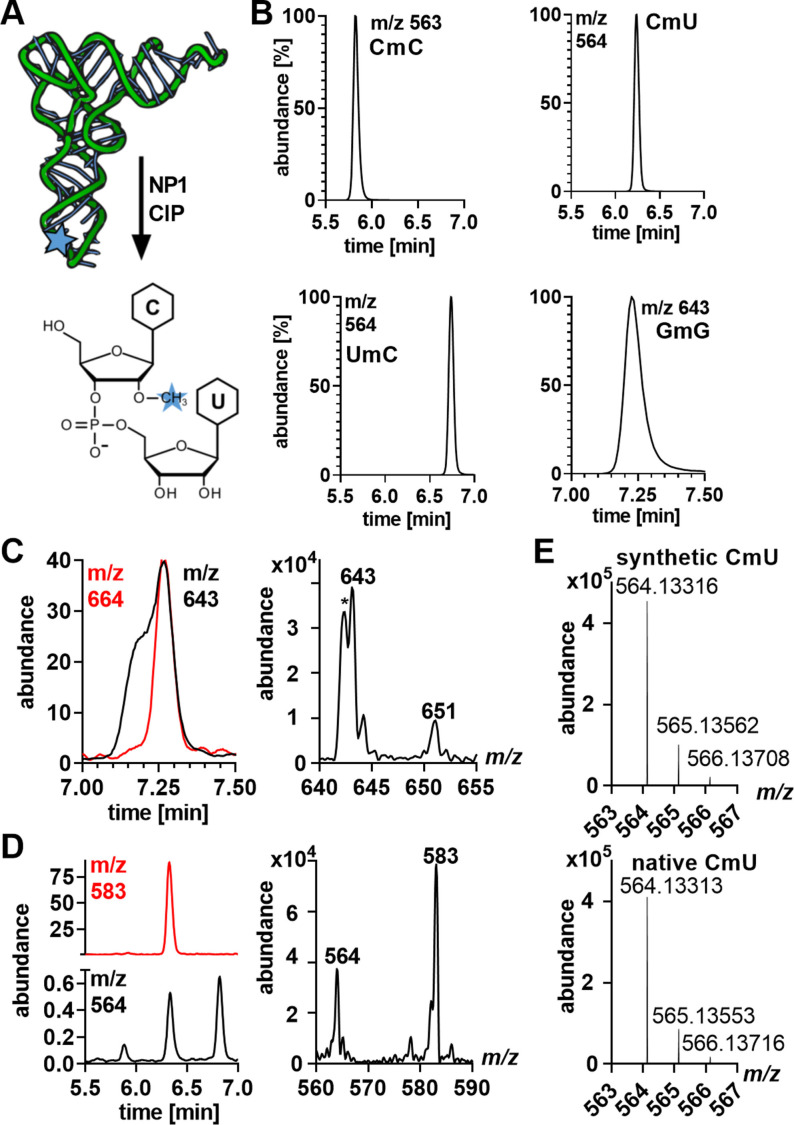
Verification of dinucleotide structures found in native RNA from *E. coli* and HEK cells. (A) Dinucleotide structure predicted through incomplete RNA hydrolysis. (B) Retention time and *m*/*z* of synthetic 2′‐*O*‐methylated dinucleotides. (C) LC‐MS/MS co‐elution of co‐injected synthetic GmG (black; *m*/*z* 643) and hydrolyzed ^13^C‐labeled RNA from *E. coli* (red; *m*/*z* 664). The MS spectrum taken from the indicated region shows the signals of the expected isotopomers. (D) Co‐injection of synthetic (black) CmU, CmC and UmC and hydrolyzed ^13^C‐labeled *E. coli* RNA (red) reveals co‐elution of one compound. (E) High‐resolution mass spectrum of synthetic and native CmC.

These results show that 2′‐*O*‐methylribonucleosides are recalcitrant to release from RNA during hydrolysis, which raises the question of the identity of the PT mimics as 2′‐*O*‐methylated dinucleotides that arise due to incomplete RNA hydrolysis. We next defined the structure of the PT mimics. To confirm the predicted structure, we synthesized 2′‐*O*‐methylated dinucleotides CmC, CmU, UmC and GmG (example given in Figure 5 A), which we used to start the workflow in Scheme 1 by first confirming the HPLC retention time of synthetic and native PT mimics (Figure 5 B). The synthetic CmC, CmU, UmC and GmG were then co‐injected with fully hydrolyzed (NP1+PDE1+CIP) ^13^C‐labeled *E. coli* RNA for LC‐MS/MS analysis, which revealed co‐elution of the GmG (*m*/*z* 643) with a molecule with *m*/*z* 664 (Figure 5 C) and CmU (*m*/*z* 564) co‐eluting with a molecule with *m*/*z* 583 (Figure 5 D, S6). The mass differences between native and isotope labeled molecules are consistent with the number of carbons in CmC, CmU, UmC and GmG fully labeled with ^13^C. High‐resolution fragmentation spectra of synthetic and putative native CmU in Figure 5 E show a molecular ion (*m*/*z* 564.133) and fragments that differ by <1 ppm (Table S1). Similar results were obtained by co‐injection with stable isotope labeled RNA from HEK cells (Figure S6).

To further confirm the identity of the 2′‐*O*‐methylated dinucleotides, we compared MS/MS signal intensities associated with mass transitions corresponding to PT and 2′‐*O*‐methylated dinucleotides. The CmU and GmG dinucleotides isolated from *E. coli*, *S. cerevisiae*, and HeLa cells showed a ≈25‐fold increase in abundance when detected with the mass transitions for the 2′‐*O*‐methylated dinucleotides rather than the PTs (Figure S7). This observation suggests that the signals detected with the PT transitions likely represent low abundance isotopomers of the dinucleotides with a mass that agrees with the mass of the respective PT dinucleotide. These results prove that 2′‐*O*‐methylated dinucleotides account for the signals described by Wu et al.^[2]^

**Figure 6:**
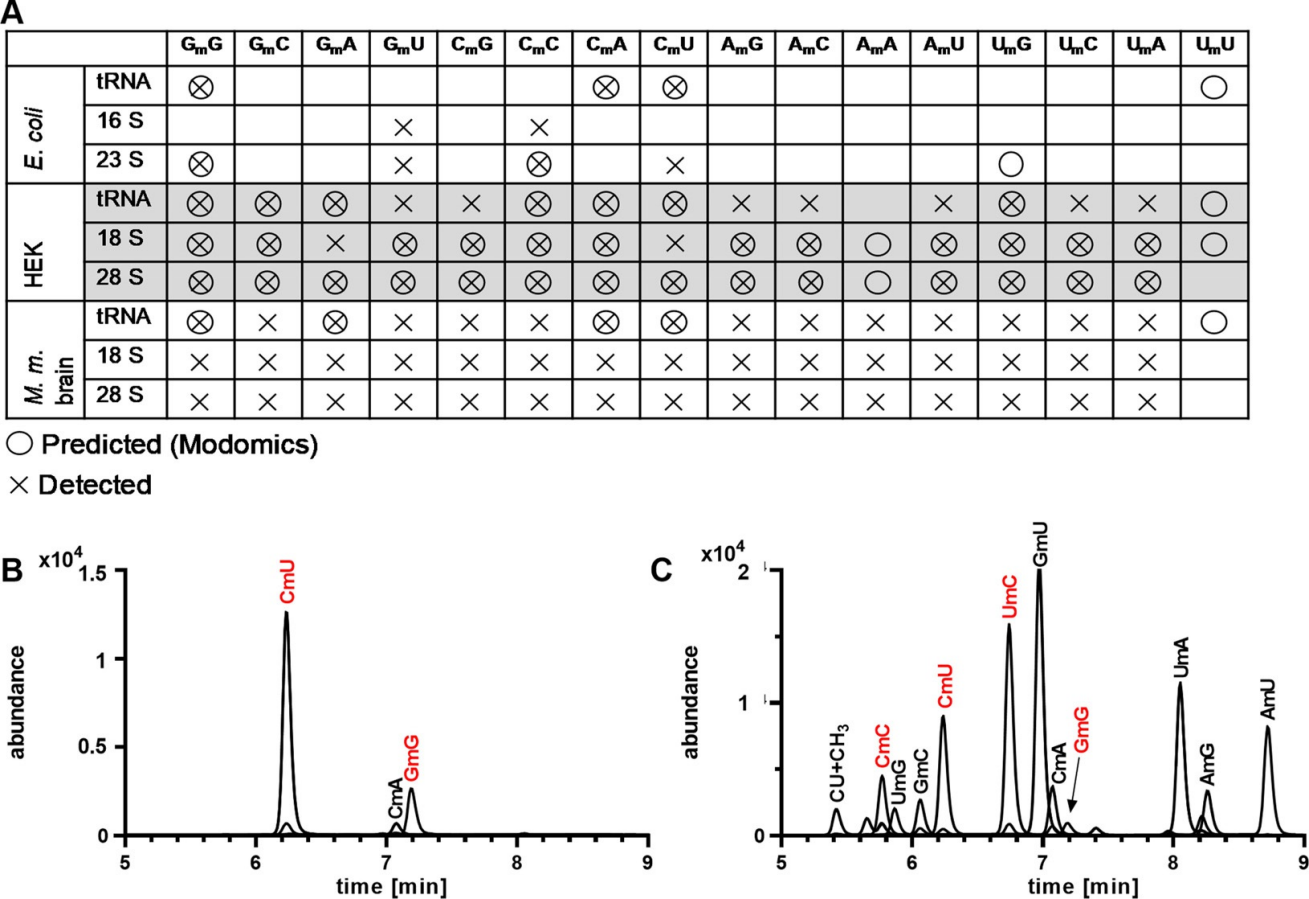
Screening for 2′‐*O*‐methylated diribonucleotides in various organisms and types of RNA. (A) 2′‐*O*‐Methylated dinucleotides predicted to occur according to literature^[1]^ and detected here by LC‐MS/MS. Composite LC‐MS chromatograms of 2′‐*O*‐methylated dinucleotides in (A) *E. coli* tRNA and (B) HEK total RNA. 2′‐*O*‐Methylated dinucleotides confirmed with synthetic standards are indicated with red font. Small signals underlying signals such as CmU hail from the M+1 signal of CmC.

2′‐*O*‐Methylation is an abundant modification in both ribosomal RNA (rRNA) and tRNA.^[1]^ Given their resistance to hydrolysis and our focus on 4 of 16 possible dinucleotide contexts, we wondered about the diversity of 2′‐*O*‐methylated dinucleotides in different organisms and different types of RNA. Literature precedent provided guidance on established dinucleotide contexts in tRNA and large and small rRNAs, as indicated by circles in Figure 6 A. Optimal hydrolysis conditions resulted in detection of only 3 of the 16 possible dinucleotide contexts in tRNA from *E. coli* (GmG, CmA and CmU; Figure 6 A,B), which contrasts with 14 detected 2′‐*O*‐methylated dinucleotides in tRNA from HEK cells, including 7 previously unreported dinucleotide sequence contexts (Figure 6 A,C). For *E. coli* 16S and 23S rRNA, we detected the reported GmG and CmC dinucleotides as well as unreported GmU and CmU contexts. We extended these studies to mouse brain RNAs for which there is little information about 2′‐*O*‐methylated dinucleotides. As shown in Figure 6 A, mouse tRNA, and 18S and 28S rRNAs possess every possible dinucleotide sequence context, including the AmA not detectable in human tRNA. These results point to the power of rigorous LC‐MS to discover new modifications and their sequence contexts. However, there are also serious limitations for interpreting the biological meaning of the LC‐MS observations. For example, while UmU has been observed in published studies,^[1]^ we were not able to detect it in any type of RNA from any organism tested (Figure 6 A). Was our inability to detect UmU and other published 2′‐*O*‐methylated dinucleotide contexts due to limited sensitivity of our instruments for rare dinucleotide motifs as well as the potential for inefficient release during hydrolysis? We are confident in the rigorous identification and quantification of those modifications that we are able to detect but those that we cannot detect cannot be ruled out and we must use orthogonal methods such as RiboMethSeq and other techniques that exploit the biochemical properties of 2′‐*O*‐methylation modifications in RNA.^[24]^

## Conclusion

The search for new post‐transcriptional RNA modifications is an important aspect of modern epitranscriptome research and mass spectrometry is the instrument of choice for the challenge. Following an established pathway for defining and validating molecular structures (Scheme 1), we discovered that the putative PT‐containing dinucleotides observed in RNA from diverse organisms^[2]^ were actually 2′‐*O*‐methylated dinucleotides. This is not the first instance of confusion about RNA modifications.[^14^, ^17^] The major sources of confusion that likely led to the misidentification appear to be incomplete hydrolysis of RNA and reliance on low‐resolution mass spectrometry. With regard to hydrolysis, we found that a minimal combination of PDE1 with either Benzonase or NP1 is required, with prolonged incubation with high nuclease concentrations providing what appears to be optimal hydrolysis of RNA to the mononucleotide level. Given published studies[^18c^, ^19^] and our observations, even with these precautions, there may be modifications that are substantially resistant to release by nuclease hydrolysis, that are released in low abundance, or that are poorly detected by mass spectrometry. These limitations demand caution in the interpretation of mass spectrometric studies of the epitranscriptome: the absence of signal does not mean the absence of the analyte.

With regard to isotopomer confusion, the M+2 signal for the abundant 2′‐*O*‐methylated dinucleotides is relatively strong and could easily be mistaken for the molecular ion M of another molecule. As illustrated in Figure 5 E, HRMS of CmU shows an isotope envelope of M of 564, M+1 of 565, and M+2 of 566, with the integer difference in *m*/*z* value validating the expected ion charge of +1. This is a very common problem that we have experienced in discovering 7‐deazaguanine modifications in DNA, with initial prediction of 2′‐deoxy‐5‐carboxy‐7‐deazaguanosine associated with *m*/*z* 311 proving to be the M+1 isotopomer of 2′‐deoxy‐7‐amido‐7‐deazaguanosine, with the error caught during rigorous structural validation studies.^[23]^ Here, the case of GmG illustrates what we suspect is the problem for misidentification of RNA PTs. The isotope envelope for the abundant GmG is comprised of M of 643, M+1 of 644, and M+2 of 645, with the putative GpsG having M of 645. The cautionary conclusion is that rigorous identification of molecular structure by mass spectrometry requires systematic exploration of all adjacent signals by full mass spectra or even HRMS to define the correct precursor molecular ion. Even now, we cannot rule out the presence of PT modifications in some type of RNA in some organism. Since we discovered PTs as natural products in DNA,^[3]^ we hope that the search continues for PTs in RNA.

## Conflict of interest

The authors declare no conflict of interest.

## Supporting information

As a service to our authors and readers, this journal provides supporting information supplied by the authors. Such materials are peer reviewed and may be re‐organized for online delivery, but are not copy‐edited or typeset. Technical support issues arising from supporting information (other than missing files) should be addressed to the authors.

Supporting Information
