# Supplementary material for: Strategies to Avoid Artifacts in Mass Spectrometry‐Based Epitranscriptome Analyses
Source: Angew Chem Int Ed Engl. 2021 Sep 29;60(44):23885–93. doi: 10.1002/anie.202106215 (PMC8597057; doi:10.1002/anie.202106215)
Supplement: Supplementary file 1 — Supporting Information [file ANIE-60-23885-s001.pdf]

Angewandte Chemie
Eine Zeitschrift der Gesellschaft Deutscher Chemiker

Supporting Information

Strategies to Avoid Artifacts in Mass Spectrometry-Based Epitranscriptome Analyses

Steffen Kaiser[+], Shane R. Byrne[+], Gregor Ammann, Paria Asadi Atoi, Kayla Borland, Roland Brecheisen, Michael S. DeMott, Tim Gehrke, Felix Hagelskamp, Matthias Heiss, Yasemin Yoluç, Lili Liu, Qinghua Zhang, Peter C. Dedon, Bo Cao, and Stefanie Kellner**

anie_202106215_sm_miscellaneous_information.pdf

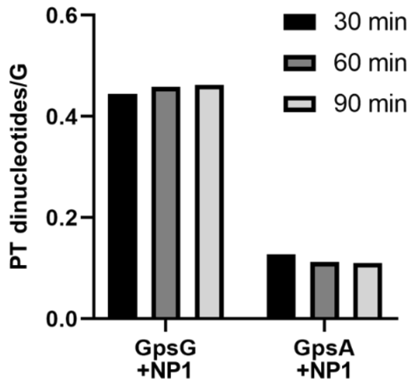

Figure S1. Synthetic PT RNA (CCCGpsGUUUA and CCCGpsAUUUA) are stable towards NP1 treatment.

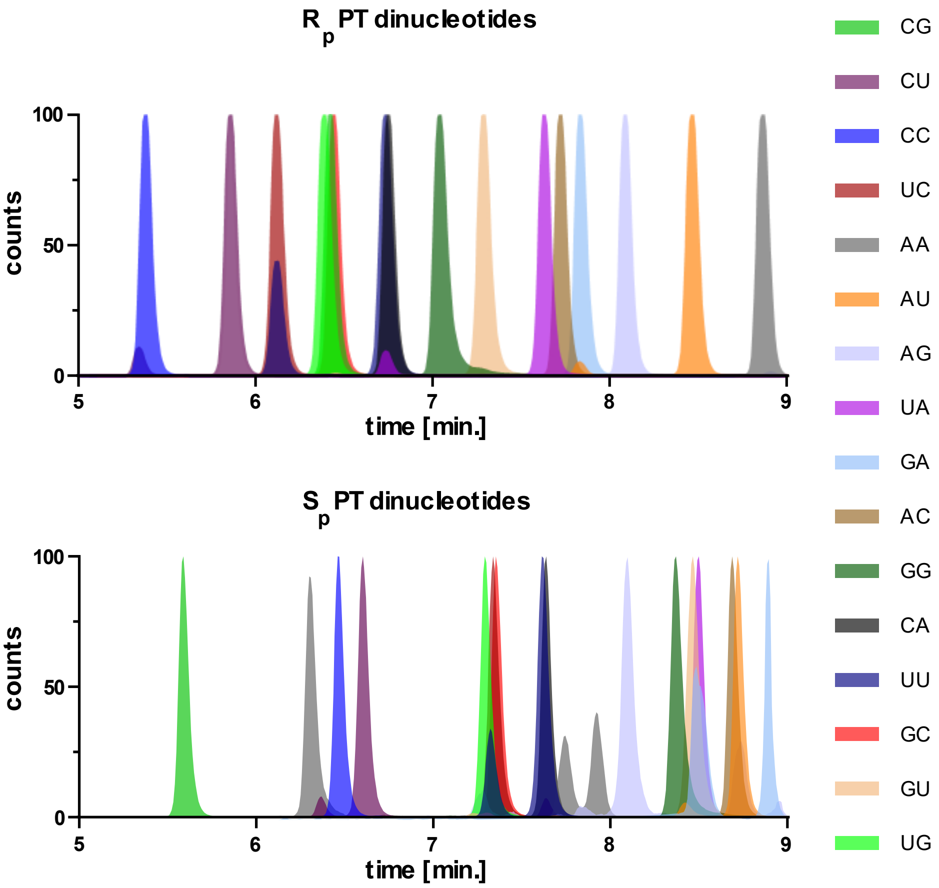

retention time [min.]		
	R_p	S_p
CC	5.4	6.5
CU	5.9	6.7
CG	6.5	5.7
CA	6.8	7.7
UC	6.2	7.4
UU	6.8	7.7
UG	6.5	7.3
UA	7.7	8.6
GC	6.5	7.5
GU	7.4	8.6
GG	7.1	8.4
GA	7.9	9.0
AC	7.8	8.8
AU	8.5	8.8
AG	8.1	8.1
AA	8.9	6.3

Figure S2. Synthetic PT diribonucleotides were synthesized following published protocols. The retention time of all 16 possible permutations for the Rp and Sp variant were recorded by KL and are shown in the chromatogram and a table for convenience.

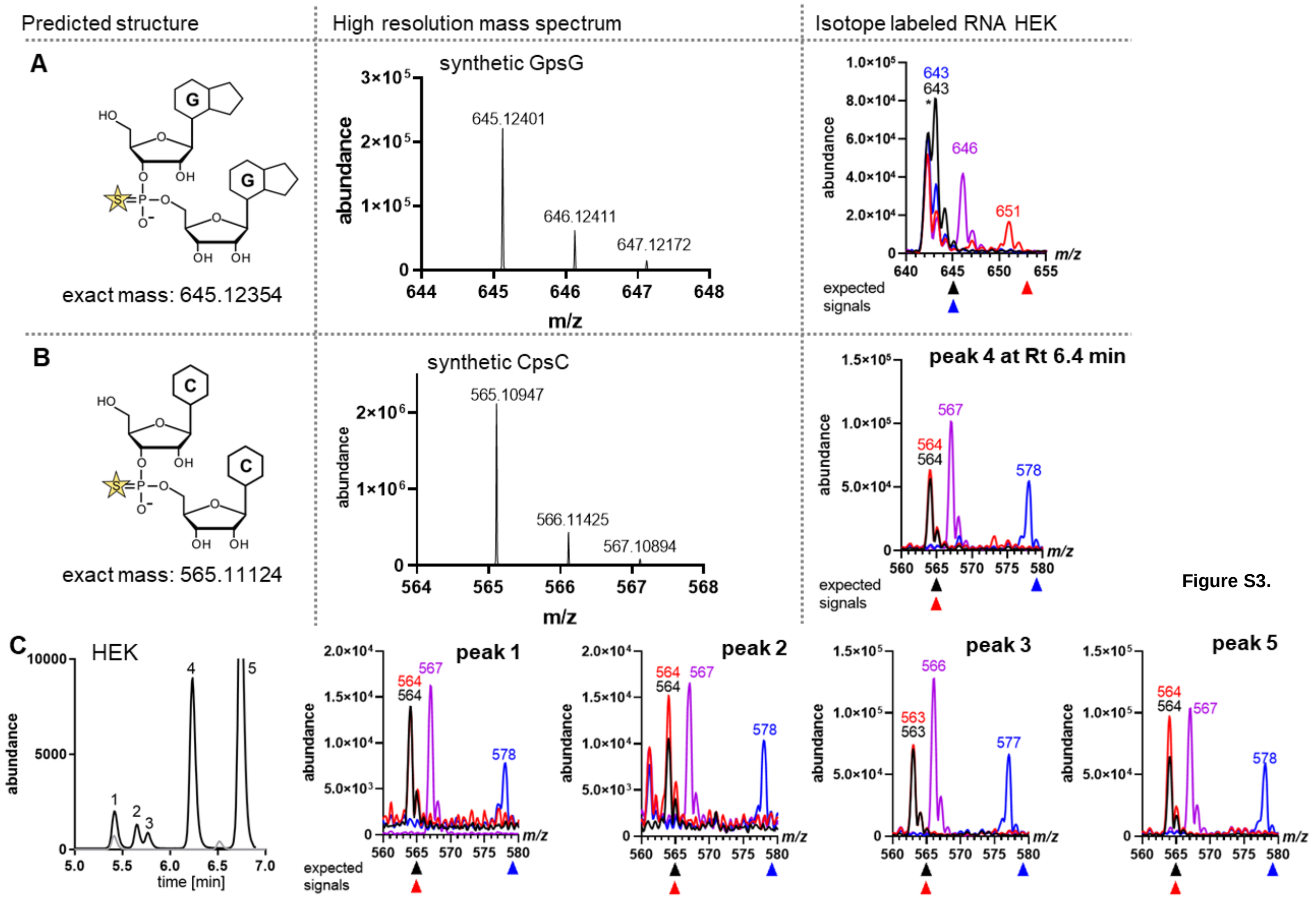

Figure S3.

Figure S3. Structure validation of native RNA PT dinucleotides GpsG and CpsC. High resolution mass spectra (HRMS) of synthetic dinucleotide precursor ions GpsG (**A**) and CpsC (**B**). Metabolically stable isotope labeled RNA from HEK cells reveals the base composition of the PTdinucleotide candidates GpsG (**A**) and CpsC (**B** peak 4 at Rt=6.4 min). Color code: black - unlabeled; red - pyrimidine labeled (+4 per guanine, +5 per adenine); blue - purine labeled (+7 per purine) and purple - L-methionine-[$^2H_3$]methyl labeled. **C** MS/MS chromatogram for targeted detection of CpsC in HEK total RNA and mass spectra of peaks 1-3 and 5 from metabolically stable isotope labeled RNA isolated from HEK. *co-eluting contaminant

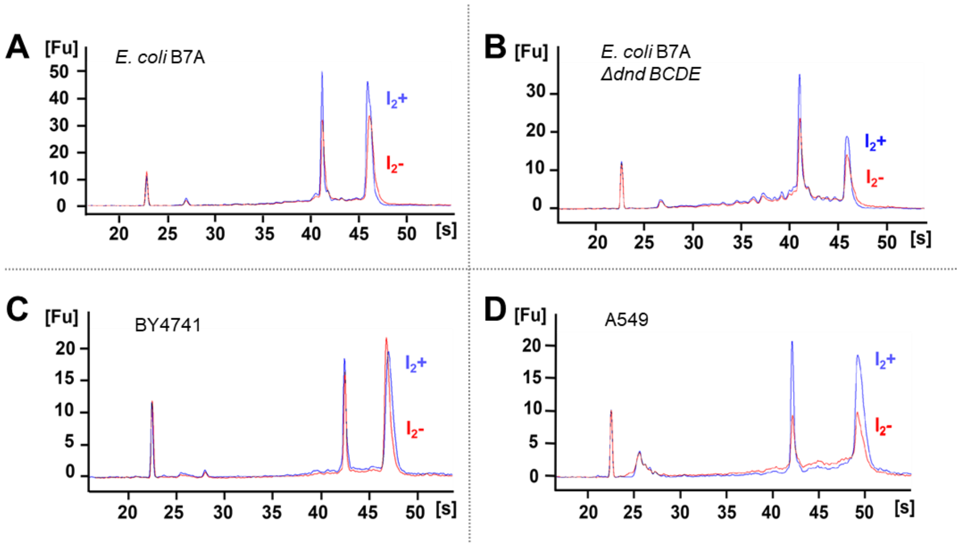

Figure S4. Bioanalyzer analysis of the integrity of total RNA from *E. coli* B7A (**A**), *E. coli* B7A (*Δdnd BCDE*) DNA PT-deficient mutant (**B**), *Saccharomyces cerevisiae* BY4741 (**C**) and human A549 cell line (**D**) after iodine cleavage of PT.

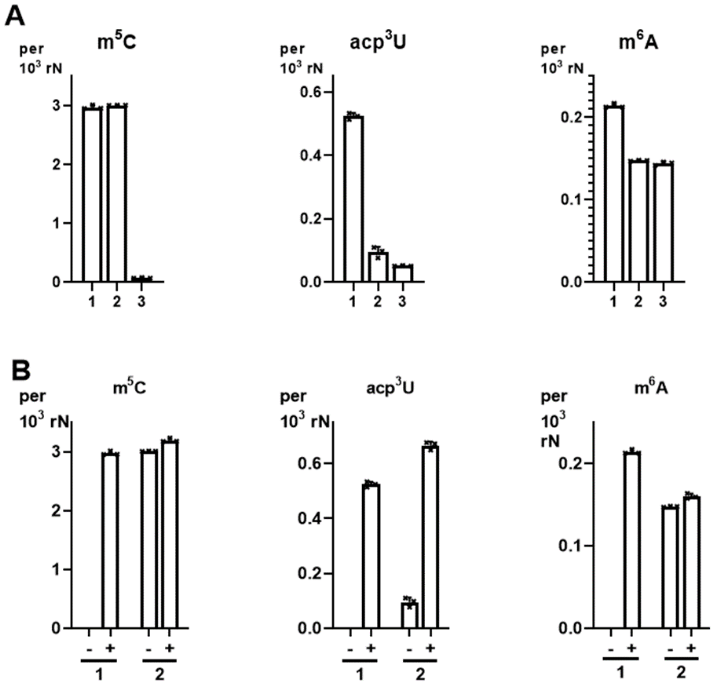

Figure S5. **A** Abundance of various modified nucleosides from HEK total RNA digested with (**1**) benzonase/PDE1/CIP [1] (**2**) NP1/CIP [2] or (**3**) a commercial RNA hydrolysis kit (NEB, Nucleoside digestion kit). **B** Abundance of various modified nucleosides from HEK total RNA digested in the absence (-) and presence (+) of phosphodiesterase 1 (PDE1) using either (**1**) benzonase+CIP [1] or (**2**) NP1+CIP [2]. All analyses from n=3 replicates, error bars represent standard deviation.

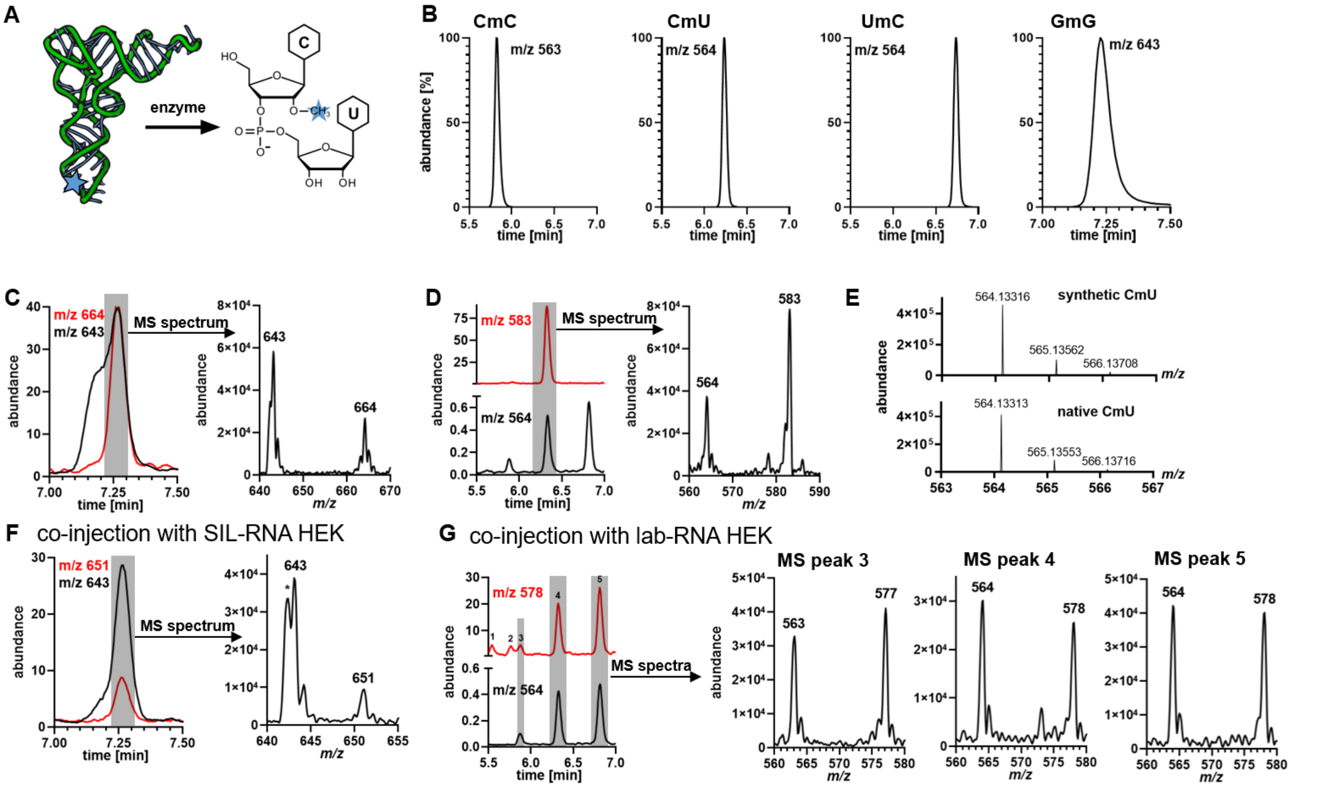

Figure S6. Verfication of dinucleotide structures found in native RNA from *E. coli* K12 and HEK cells. **A** Release of 2'-O-methylated dinucleotides by optimized digestion parameters. **B** Retention time of synthetic GmG, CmC, CmU and UmC dinucleotides. **C** and **D** co-injection of native ribodinucleotides (from *E. coli* K12, ^{13}C-labeled) and synthetic GmG (C) and CmU (D) **E** High-resolution MS of synthetic and native CmU. **F** and **G** co-injection of synthetic (black) GmG (**F**) and CmU, CmC and UmC (**G**) and stable isotope labeled (SIL) RNA from HEK cells (red) reveals co-elution. The MS spectra taken from the indicated peaks show the signals of the expected isotopologues. Peak 3: CmC, peak 4: CmU and peak 5: UmC.

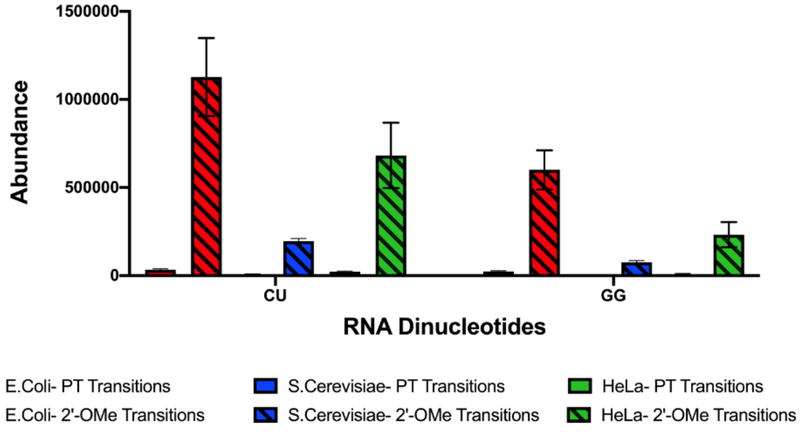

Figure S7. Quantification of the RNA dinucleotides in RNA isolated from *E. coli* B7A, *S. cerevisiae*, and HeLa cells. The peak abundances were determined using either the mass transitions corresponding to the PT dinucleotides or the 2'-OMe dinucleotides on an Agilent 6490 LC-QQQ-MS. The following transitions, were monitored: 566.1→112.1, 564.1→112.1, 645.1→152.1, 643.1→152.1.

Table S1. High-resolution mass spectrometric data of synthetic RNA PT diribonucleotides, synthetic 2'-O-methylated diribonucleotides and native „unknown" dinucleotide signals from *E. coli* B7A and *S.cerevisiae*.

Dinucleotide	Calculated Mass	Observed Mass	Mass Difference	Retention Time (min)
CC PT	565.11124	565.11053	0.00071	2.00
CU PT	566.09525	566.09363	0.00162	2.39
GG PT	645.12354	645.12213	0.00141	4.47
UC PT	566.09525	566.09467	0.00058	2.79
CmC	563.14973	563.14825	0.00148	2.13
CmU	564.13375	564.13318	0.00057	2.82
GmG	643.16203	643.16058	0.00145	5.10
UmC	564.13375	564.13294	0.00081	3.51
CU unknown E.Coli B7A	564.13375	564.13446	-0.00071	2.89
GG unknown E.Coli B7A	643.16203	643.16144	0.00059	5.06
CU unknown S.Cerevisiae	564.13375	564.13446	-0.00071	2.88
GG unknown S.Cerevisiae	643.16203	643.16101	0.00102	5.11

Materials & Methods

The data presented in this manuscript was prepared by three labs independently of each other. Whenever similar but at crucial steps different materials or methods were used, the respective lab is indicated. The following abbreviations are used: CL (Cao lab), DL (Dedon lab) and KL (Kellner lab)

General Materials. Nuclease P1 was purchased from either US Biological (DL) or Sigma-Aldrich (KL) and reconstituted according to the manufacturer's protocols. Calf intestinal alkaline phosphatase was purchased from Sigma. The enzyme Nucleoside Digestion Mix was purchased from New England BioLabs (Ipswich, MA; CL and KL). RNA and DNA

oligonucleotides containing PTs were purchased from Integrated DNA Technologies (IDT, DL), Ella Biotech (Munich, Germany, KL) or Sangon Biotech Co. Ltd. (Shanghai, Cao lab). *Escherichia coli (E. coli)* B7A and *Saccharomyces cervisiae (S. cerevisiae)* were prepared as described previously [3]. The *E.coli* B7A PT deficient mutant *(ΔdptB-H)* was from our previous study [3]. The A549 cell line was obtained from Pro. Yunfeng Zhao (Qufu Normal University, China; CL). HEK 293 cells were obtained from DSMZ (KL). TRI Reagent and D-(+)-glucose were purchased from Sigma. The RNA isolation kit RNAiso Plus was purchased from Takara, RNAprep Pure Cell/Bacteria Kit from TIANGEN (Beijing China) and Oligo Clean & Concentrator™ was from ZYMO REASEARCH (Irine, CA) (CL). The PureLink miRNA Isolation Kit was purchased from ThermoFisher (DL). BD Difco LB Broth, Bacto Yeast Extract, Bacto Agar, and Bacto Peptone were purchased from Fisher Scientific (DL). Spin filters (10 kDa) were purchased from VWR Sigma.

Cell culture and RNA isolation. A single colony of *E. coli* B7A was grown in 5 mL of LB medium (1% tryptone, 0.5% yeast extract, and 1% sodium chloride) overnight at 37 °C. The cell culture was aliquoted in 1 mL increments into 2 mL microcentrifuge tubes. The cells were harvested by centrifuging at 4000 rpm at 4 °C for 10 min. 1 mL of TRI reagent was added to each cell pellet and the total RNA isolated according to manufacturer's protocol (DL and KL). Instead of immediate harvest of overnight cultures, CL inoculated the seed medium in 10 ml of fresh LB medium according to the 0.1% inoculum, followed by growth at 37℃, 220 rpm until OD_{600}=0.6-0.8. Culture pellets were harvested by centrifugation at 12000 rpm for 2 min at 4℃ and immediately used for total RNA extraction using the RNAprep Pure Cell/Bacteria Kit (TIANGEN) following the manufacturer's protocol (CL).

A single colony of *S. cerevisiae* was grown in 5 mL of YPD medium (1% yeast extract, 2% peptone, 2% dextrose) overnight at 30 °C with shaking at 170 rpm. The cell culture was aliquoted in 1 mL increments into 2 mL microcentrifuge tubes. The cells were harvested by centrifuging at 1500 rpm at 4 °C for 10 min. The pellet was washed twice with RNase-free H_2O. The cell pellets were resuspended in 1 mL TRI Reagent and homogenized using a FastPrep homogenizer at 4 °C using a setting of 4.0 for 30 s and repeated for a total of two cycles. The cells were incubated on ice for 10 min, before the addition of 200 µL of chloroform. The mixture was vortexed for 1 min, incubated on ice for 1 min, and centrifuged at 12,000 g at 4 °C for 15 min. The upper aqueous layer (~400 µL) was transferred to a new 2 mL microcentrifuge tube and ethanol (915 µL) was added to precipitate the RNA. The mixture was loaded onto a PureLink spin cartridge and centrifuged at 12,000 g at 4 °C for 1 min. The column was washed twice with wash buffer W5 (500 µL) and centrifuged at 12,000 g at 4 °C for 1 min. The spin cartridge was then centrifuged at 12,000 g at 4 °C for 1 min to remove residual wash buffer. The RNA was eluted with 100 µL of H_2O by centrifuging at 12,000 g at 4 °C for 1 min (DL).
Instead of immediate harvest of overnight culture, CL inoculated the seed medium in 10 ml of fresh YPD medium according to the 0.1% inoculum, followed by growth at 30℃, 220 rpm until OD_{600}=0.6-0.8. Culture pellets (no more than 2 x 10^7 cells) were harvested by centrifugation at 12000 rpm for 2 min at 4℃ for digestion of yeast cell wall using lyticase (OMEGA). For total RNA extraction the RNAiso Plus (Takara) was immediately used according to the manufacturer's protocol (CL).
KL: All cell culture media and supplements were obtained from Sigma-Aldrich (Munich, Germany) unless stated otherwise. Standard Basal medium for HEK 293 culture was DMEM D6546 high glucose supplemented with 10% FBS and 0.584 g/L L-glutamine. Cells were split 1:7 using standard procedures every 2-3 days to counter overgrowth. Cells cultured in DMEM medium were kept at 10% CO_2 for proper pH adjustment. For all experiments where labeling

of nucleosides was involved DMEM D0422 without methionine and cystine was used. DMEM D0422 was supplemented with 10% dialyzed FBS (Biowest, Nuaillé, France), 0.584 g/L L-glutamine, 0.063 g/L cystine (stock concentration 78.75 g/L dissolved in 1M HCl), 0.03 g/L methionine, 0.05 g/L uridine and 0.015 g/L adenine. Uridine, adenine and methionine were either added as unlabeled or labeled compounds depending on the desired labeling [4]. $^{13}C_5$,$^{15}N_2$-Uridine (Ribose-$^{13}C_5$, 98%; $^{15}N_2$, 96-98%) and $^{15}N_5$-Adenine ($^{15}N_5$, 98%) were obtained from Cambridge Isotope Laboratories (Tewksbury, MA, USA). HeLa cells were cultured and labeled using the same media.

A549 cells were cultured in RPMI-1640 medium (Gibco) with 10% fetal bovine serum with 5% CO_2 at 37℃. Cells were harvested at 70-90 % confluency by addition of 0.25% (w/v) Trypsin and 0.53 mM EDTA (Gibco) solution at 37℃ to barely coat cells for 5 minutes at room temperature. Total RNA from the collected cells was extracted using RNAiso Plus according to the manufacturer's protocol (CL).

RNA purification. tRNA and rRNA subunits were purified from total RNA using size exclusion chromatography following established protocols [5].

Hydrolysis of RNA for LC-MS analysis. Synthetic PT containing oligonucleotides and native RNA were hydrolyzed using enzymatic protocols as indicated in figure legends.
Nuclease P1 (NP1): Hydrolysis with NP1 was performed by KL (Figure 1, Figure 2 isotope labeled RNA, Figures 4-6) as follows: 1 µg of RNA was incubated with 1 U NP1 and 1 U calf intestinal alkaline phosphatase (CIP) in 50 mM ammonium acetate pH 5.3, 0.5 mM $ZnCl_2$ and 10 µM Tris-HCl (pH 8 20 µL total reaction volume) at 37°C for 30 minutes or as indicated. The DL (Figure 2 UHPLC-MS and HRMS) used a two pot variant of the above protocol: Total RNA (20-40 µg, 78 µL) was incubated with NP1 (1.5 U, 3 µL) in 30 mM ammonium acetate pH 5.3 and 0.5 mM $ZnCl_2$ (90 µL total reaction volume) for 2 h at 55 °C. The reaction mixture was diluted with Tris-HCl (10 mM) final concentration, pH 8.0, 9 µL) and incubated with CIP (51 U, 3 µL) for 2 h at 37 °C. Enzymes were removed by passing the mixture through a VWR 10 kDa spin filter with centrifugation at 12,000 g for 12 min. The solution was lyophilized to dryness and resuspended in H_2O (50 µL) (DL).
Benzonase: RNA (300 ng) in aqueous digestion mix (30 µL) was digested to single nucleosides by using 2 U alkaline phosphatase, 0.2 U phosphodiesterase I (VWR, Radnor, Pennsylvania, USA), and 2 U benzonase in Tris (pH 8, 5 mM) and $MgCl_2$ (1 mM) containing buffer. Furthermore, 0.5 µg tetrahydrouridine (Merck, Darmstadt, Germany), 1 µM butylated hydroxytoluene, and 0.1 µg pentostatin were added to avoid deamination and oxidation of the nucleosides. When quantification of dihydrouridine was intended tetrahydrouridine was omitted. After incubation for 2 h at 37 °C, 20 µL of LC-MS buffer A (QQQ) was added to the mixture and then filtered through 96-well filter plates (AcroPrep Advance 350 10 K Omega, PALL Corporation, New York, USA) at 3000 ×g and 4 °C for 30 min. A stable isotope labeled internal standard (SILIS) was produced in *S. cerevisiae* using ^{13}C and ^{15}N rich growth medium (Silantes, Munich, Germany, Product# 111601402) following recently described procedures [6]. 1/10 Vol. of SILIS was added to each filtrate before analysis by QQQ mass spectrometry. For each sample 10 µL were injected (~90 ng of sample RNA)
Commercial hydrolysis protocol: 1 µg of native RNA (KL) and iodine treated RNA (CL), were digested by Nucleoside Digestion Mix (NEB, M0649S) following manufacturer’s protocol. CL used Amicon® Ultra-0.5 filtration centrifuge tubes (MERCK MILL IPORE) for filtration and digestion products were lyophilized and reconstituted in 10 µL of RNase-free water (CL).

LC-MS/MS analysis of RNA dinucleotides. Core instrumentation and parameters used for RNA hydrolysate analysis by the DL, CL and KL are displayed in the table below.

Supplementary Table S2: Instrumentation and parameters used for targeted RNA modification analysis

	KL	DL	CL
(U)HPLC	Agilent 1290 series HPLC equipped with diode array detector	Agilent 1290 series HPLC equipped with diode array detector	Sciex EXION LC System
Mass spectrometer	Agilent 6470 triple quadrupole	Agilent 6490 triple quadrupole	SCIEX 6500 QTRAP+triple quadrupole
Column	Synergi Fusion RP column (2.5 µm particle size, 100 Å pore size, 100 mm length, 2 mm inner diameter) Phenomenex, Aschaffenburg, Germany		Waters ACQUITY UPLC HSS T3 column (100 × 2.1 mm, 1.7 *µ*m)
Buffer A	5 mM ammonium acetate pH 5.3		
Buffer B	acetonitrile		
Flow rate	0.35 ml/min		0.35 mL/min
Column temp.	35 °C		
Gradient	Start: 0% B<br>0-1’: 0% B<br>1-8’: 10% B<br>8-10’: 40% B<br>10-11’: 40% B<br>11.5’: 0% B<br>2.5’ for regeneration	Start: 3% B<br>0-15’: 9% B<br>15-16’: 95% B<br>Rinse with 3% B for 3’ for regeneration	Start: 1% B<br>0-6’: 1-12% B<br>6-6.1’: 99% B<br>6.1-7.1’: 99% B<br>Rinse with 1% B for 3’ for regeneration
ESI parameters			
N_2 temperature	230 °C	200 °C	IonDrive Turbo V electrospray ionization: Curtain Gas = 40 psi, IonSpray Voltage = ±4500 V, temperature = 500 ℃, Ion Source Gas 1 = 30 psi, Ion Source Gas 2 = 30 psi
N_2 flow rate	6 L/min	14 L/min	
nebulizer pressure	40 psi	20 psi	
capillary voltage	2500 V	1800 V	
fragmentor voltage	As indicated in tables	380 V	
ionization mode	positive	positive	positive
retention times R_t	See Tables S3 and S4		

MS scan was performed in the range of m/z 550-700 with a 150 ms Scan time (Fragmentor Voltage 120 V) by KL.
Targeted analyses in multiple reaction monitoring mode (MRM = MS/MS) was done by KL and DL lab using the parameters indicated in Tables S3, S4 and S5)

Supplementary Table S3: MS/MS parameters for detection of **PT dinucleotides** from unlabeled, $^{13}C_6$-glucose labeled, ^{15}N-ammonia labeled, and ^{34}S-sulfate labeled *E. coli* K12 cultures (KL). Cell Accelerator Voltage was kept at 5V for all compounds.

	**unlabeled**		**13C**		**15N**		**34S**					
	Precursor Ion	Product Ion	Precursor Ion	Product Ion	Precursor Ion	Product Ion	Precursor Ion	Product Ion	Ret Time (min)	Delta Ret Time	Fragmentor Voltage (V)	Collision Energy (eV)
AA	613	136	633	141	623	141	615	136	8,9	2	120	40
AC	589	136	608	141	597	141	591	136	7,8	2	120	40
AG	629	136	649	141	639	141	631	136	8,1	2	120	40
AU	590	136	609	141	597	141	592	136	8,5	2	120	40
CA	589	136	608	141	597	141	591	136	6,8	2	120	40
CC	565	112	583	116	571	115	567	112	5,4	2	120	40
CG	605	152	624	157	613	157	607	152	6,5	2	120	40
CU	566	112	584	116	571	115	568	112	5,9	2	120	40
GA	629	136	649	141	639	141	631	136	7,9	2	120	40
GC	605	152	624	157	613	157	607	152	6,5	2	120	40
GG	645	152	665	157	655	157	647	152	7,1	2	120	40
GU	606	152	625	157	613	157	608	152	7,4	2	120	40
UA	590	136	609	141	597	141	592	136	7,7	2	120	40
UC	566	112	584	116	571	115	568	112	6,2	2	120	40
UG	606	152	625	157	613	157	608	152	6,5	2	120	40
UU	567	113	585	117	571	115	569	113	6,2	2	120	40
A	268	136	278	141	273	141	268	136	6	2	250	25
C	244	112	253	116	247	115	244	112	2,5	2	250	20
G	284	152	294	157	289	157	284	152	5	2	250	25
U	245	113	254	117	247	115	245	113	3	2	100	20

Supplementary Table S4: MS/MS parameters for detection of 2'-O-methylated dinucleotides from unlabeled RNA (KL). Cell Accelerator Voltage was kept at 5V for all compounds.

	**unlabeled**					
Compound Name	Precursor Ion	Product Ion	Ret Time (min)	Delta Ret Time	Fragmentor Voltage (V)	Collision Energy (eV)
AA	611	136	8,7	2	120	40
AC	587	136	8,2	2	120	40
AG	627	136	8,3	2	120	40
AU	588	136	8,7	2	120	40
CA	587	136	7,1	2	120	40
CC	563	112	5,8	2	120	40
CG	603	152	?	2	120	40
CU	564	112	6,35	3	120	40
GA	627	136	8	2	120	40
GC	603	152	6,1	2	120	40

GG	643	152	7,2	2	120	40
GU	604	152	7	2	120	40
UA	588	136	8,1	2	120	40
UC	564	112	7	2	120	40
UG	604	152	5,9	2	120	40
UU	565	113	?	2	120	40
A	268	136	6	2	250	25
C	244	112	2,5	2	250	20
G	284	152	5	2	250	25
U	245	113	3	2	100	20

Supplementary Table S5: Triple quadrupole MS parameters used by DL for targeted analysis of dinucleotides

**Species**	**Precursor Ion Mass**	**Product Ion Mass**	**Collision Energy (V)**	**Cell Accelerator Voltage (V)**
CU PT	566.1	113.1	36	5
	566.1	112.1	28	5
GG PT	645.1	152.1	36	3
CmU	564.1	113.1	36	5
	564.1	112.1	28	5
GmG	643.1	152.1	36	3
rA	268.1	136.1	12	1
rC	244.1	112.1	16	1
rG	284.1	152.1	4	3
rU	245.1	127.1	12	1

High resolution mass spectrometry (DL). Synthetic PT or 2'-OMe RNA dinucleotides (10 pmol per 10 µL injection) or NP1/CIP hydrolyzed RNA were analyzed on a Dionex Ultimate 3000 UHPLC system coupled to a Thermo Fisher Q Exactive Hybrid Quadrupole-Orbitrap mass spectrometer. Chromatographic separation was achieved as described in Table S1. High resolution mass spectra for the PT and 2'-OMe-containing dinucleotides were obtained by hybrid quadrupole-Orbitrap mass spectrometry with the following parameters: sheath gas flow rate, 50 L/min; aux gas flow rate, 15 L/min; sweep gas flow rate, 3 L/min; spray voltage, 4.20 kV; and capillary temperature, 275 °C.

Iodine treatment of RNA oligonucleotides and analysis by HPLC (CL). Iodine treatment was performed in a 50 µL reaction system containing 10 µL of RNA oligonucleotides (20 µM, 5'- AUG CAC CUA G*GA UAU AGA ACG UAG AUA UGC -3'. (* : PT modification)), 5 µL of Tris-HCl (500 mM, pH 7.0), 5 µL of iodine solution (0.1 N, FLUKA) and RNase-free water. After incubation at 65 °C for 5 min, the reaction products were kept on ice. Followed by purification using Oligo Clean & Concentrator™ kit (ZYMO) to remove salts and iodine, the purified products were subjected to analysis by an Agilent 1260 infinity II, equipped with an Agilent Poroshell 120 EC-C18 column (3.0 × 50 mm, 2.7 µm, Agilent). The mobile phase A was 100 mM TEAA (PH 6.0), and the mobile phase B was the mixture of 100 mM TEAA and

acetonitrile (v/v=80:20). Buffer B gradient elution in 20min from 40% to 60%. The flow rate was set at 0.3 mL/min and the column temperature was set at 40℃.

Iodine treatment of total RNA and analysis by Bioanalyzer (CL). Iodine treatment was performed in a 50 µL reaction system containing 6 µg of the total RNA, 5 µL of Tris-HCl (500 mM, pH 7.0), 5 µL of iodine solution and RNase-free water. In the negative control sample, H_2O was used instead of iodine into the reaction system. After incubation at 65 °C for 5 min, kept the reaction products on ice. Followed by purification using Oligo Clean & Concentrator™ kit (ZYMO) to remove salts and iodine, the purified products were subjected to analysis by Agilent Bioanalyzer with the RNA 6000 Nano Kit according to the manufacturer's protocol.

References
[1] W. M. Cai, Y. H. Chionh, F. Hia, C. Gu, S. Kellner, M. E. McBee, C. S. Ng, Y. L. Pang, E. G. Prestwich, K. S. Lim, I. R. Babu, T. J. Begley, P. C. Dedon, *Methods Enzymol* **2015**, *560*, 29-71.
[2] P. F. Crain, *Methods Enzymol* **1990**, *193*, 782-790.
[3] B. Cao, C. Chen, M. S. DeMott, Q. Cheng, T. A. Clark, X. Xiong, X. Zheng, V. Butty, S. S. Levine, G. Yuan, M. Boitano, K. Luong, Y. Song, X. Zhou, Z. Deng, S. W. Turner, J. Korlach, D. You, L. Wang, S. Chen, P. C. Dedon, *Nat Commun* **2014**, *5*, 3951.
[4] M. Heiss, F. Hagelskamp, V. Marchand, Y. Motorin, S. Kellner, *Nat Commun* **2021**, *12*, 389.
[5] aY. H. Chionh, C. H. Ho, D. Pruksakorn, I. Ramesh Babu, C. S. Ng, F. Hia, M. E. McBee, D. Su, Y. L. Pang, C. Gu, H. Dong, E. G. Prestwich, P. Y. Shi, P. R. Preiser, S. Alonso, P. C. Dedon, *Nucleic Acids Res* **2013**, *41*, e168; bF. Hagelskamp, K. Borland, J. Ramos, A. G. Hendrick, D. Fu, S. Kellner, *Nucleic Acids Res* **2020**, *48*, e41.
[6] S. Kellner, A. Ochel, K. Thuring, F. Spenkuch, J. Neumann, S. Sharma, K. D. Entian, D. Schneider, M. Helm, *Nucleic Acids Res* **2014**, *42*, e142.
